# Supervised Approach to Identify Autism Spectrum Neurological Disorder via Label Distribution Learning

**DOI:** 10.1155/2022/4464603

**Published:** 2022-08-27

**Authors:** N. V. L. M Krishna Munagala, V. Saravanan, Firas Husham Almukhtar, Naveed Jhamat, Nadeem Kafi, Samiullah Khan

**Affiliations:** ^1^Department of Electrical Electronics and Communication Engineering, GITAM Institute of Technology, GITAM Deemed University, Visakhapatnam, Andhra Pradesh 530045, India; ^2^Dambi Dollo University, Dambi Dollo, Ethiopia; ^3^Department of Computer Technical Engineering, Imam Ja'afar Al-Sadiq University, Kirkuk, Iraq; ^4^Department of Information Technology, University of the Punjab, Gujranwala Campus, Gujranwala, Pakistan; ^5^Department of Computer Science, National University of Computer and Emerging Sciences, Karachi, Pakistan; ^6^Department of Maths, Stats & Computer Science, The University of Agriculture Peshawar, Peshawar, KP, Pakistan

## Abstract

Autism Spectrum Disorder (ASD) is a complicated collection of neurodevelopmental illnesses characterized by a variety of developmental defects. It is a binary classification system that cannot cope with reality. Furthermore, ASD, data label noise, high dimension, and data distribution imbalance have all hampered the existing classification algorithms. As a result, a new ASD was proposed. This strategy employs label distribution learning (LDL) to deal with label noise and uses support vector regression (SVR) to deal with sample imbalance. The experimental results show that the proposed method balances the effects of majority and minority classes on outcomes. It can effectively deal with imbalanced data in ASD diagnosis, and it can help with ASD diagnosis. This study presents a cost-sensitive approach to correct sample imbalance and uses a support vector regression (SVR)-based method to remove label noise. The label distribution learning approach overcomes high-dimensional feature classification issues by mapping samples to the feature space and then diagnosing multiclass ASD. This technique outperforms previous methods in terms of classification performance and accuracy, as well as resolving the issue of unbalanced data in ASD diagnosis.

## 1. Introduction

Autism spectrum disorder (ASD) is a series of complex neurodevelopmental disorders, and its clinical manifestations are mainly social interaction disorders, verbal communication disorders, and stereotyped repetitive movements [1, 2]. Statistics from the US Centers for Disease Control and Prevention show that the prevalence of autism in American children is as high as 1 : 59. This shows that autism has become a rather serious health problem and there is an urgent need to develop an effective method for timely diagnosis. However, because the physiological cause of autism is not clear, medical diagnosis can only be based on the patient's symptoms and feedback, qualitative/quantitative testing information, and the physician's personal experience, which has great uncertainty [3]. Therefore, it is of great significance to use computers to assist in the diagnosis of autism.

Studies have shown that autism spectrum disorders are related to the abnormal brain function in patients and resting-state functional magnetic resonance imaging, which reflects functional changes such as brain metabolic activity in patients under a resting state, is reflected using blood oxygen-dependent levels [4, 5]. Resonance image (resting-state functional magnetic resonance imaging, rs-fMRI) has become a powerful tool for quantifying neural activity in the brain and has gradually become one of the important means for the study of brain diseases such as ASD [6, 7]. Based on this diagnosis, researchers have proposed a variety of computer-aided autism diagnosis algorithms [8, 9]. For example, the authors used high-order functional connectivity matrix for auxiliary diagnosis of autism. He proposed multivariate graph learning for auxiliary diagnosis of autism, the authors explored the relationship between brain regions through deep learning and Correlation for auxiliary diagnosis of autism and so on [10]. However, these methods can only deal with dichotomous problems, and in clinical practice, autism spectrum disorder includes several disorders related to developmental disorders, such as autism [11] and Asperger's syndrome (Asperger's disorder), nonspecific general developmental disorders (pervasive developmental disorder not otherwise specified, PDD-NOS), and so on. Most of the existing auxiliary diagnosis models for autism can only solve the problem of binary classification and cannot distinguish several related diseases of ASD at the same time. In addition, these methods also do not deal with label noise in a targeted manner [12]. Labeling noise is a challenge involved in the auxiliary diagnosis of multiclass ASD and has serious adverse effects on classifier performance [13]. Label noise refers to the deviation between the target label of the training sample and the true label of the corresponding instance. There are many factors in the generation of labeling noise, such as the subjectivity of the labeling process, the low recognizability of the samples to be labeled, and communication/coding problems. Labeling noise is prevalent in autism diagnosis scenarios. Subjectivity in the diagnostic process, inconsistent diagnostic criteria, and blurring of the boundaries of ASD subcategories contribute to labeling noise [14].

The class imbalance problem under high-dimensional features is another challenge involved in the auxiliary diagnosis of multiclass ASD [15]. The neuroimaging data usually used for the auxiliary diagnosis of ASD often have hundreds or thousands of features, and the number of training samples is very limited, which may easily lead to overfitting problems during classifier training. Moreover, the samples used to construct the ASD classifier have the problem of class imbalance, which causes the classification prediction results to be biased towards the majority class [16, 17]. This paper proposes a cost-sensitive label distribution support vector regression learning for auxiliary diagnosis of ASD [18]. First of all, multiclass ASD auxiliary diagnosis is faced with the problem of label noise, and the unique label form of label distribution can better overcome the influence of label noise on the classifier through the description of the same sample by different labels to accurately express the difference between labels. The degree of correlation makes the learning process contain richer semantic information, can better distinguish the relative importance of multiple markers, and has better pertinence to the problem of marker noise in the auxiliary diagnosis of ASD [19, 20]. The kernel approach is introduced at the same time as the support vector regression. The linearly inseparable data in the original input space may be transferred into a linearly separable feature space using the kernel method's nonlinear mapping, offering additional discriminative information. Finally, a cost-sensitive technique is devised to address the issue of category imbalance. The algorithm may adjust to the demands of actual applications to some degree and treat a limited number of individuals equitably by introducing the imbalance of misjudgment costs of various categories in reality.

Label distribution learning (LDL) is designed to cope with label noise in this technique, while support vector regression (SVR) is also used to handle the sample imbalance. According to the results of the trials, the proposed technique optimizes the effects of majority and minority classes on outcomes. It can handle skewed data in ASD diagnosis and can assist with ASD diagnosis. This study provided a cost-sensitive technique for correcting sample imbalance using a support vector regression (SVR)-based method to reduce label noise. The label distribution learning approach addresses high-dimensional feature classification challenges by mapping data to the feature space and then diagnosing multiclass ASD. In terms of classification performance and accuracy, our proposed strategy outperforms earlier methods, as well as eliminates the challenge of unbalanced data in ASD diagnosis.

However, the improved model is still biased towards the majority class to some extent, and the imbalanced data problem should be improved further as a future study. Researchers can further try to improve the data sampling method or use the synthetic minority sample method as future prespective

### 1.1. Organization

The paper is framed into several sections where Section 1 states about the Introduction followed by related work section in Section 2.  Section 3 states about cost-sensitive marker distribution learning for ASD-aided diagnosis, followed by Section 4 that describes the evaluation of proposed methodology. The final section is the concluding section numbered 5 that discusses the results obtained in the study.

## 2. Related Work

### 2.1. Labeled Distribution Learning

Label distribution learning (LDL) is a machine learning method that has emerged in recent years [21]. It introduces the concept of label distribution on the basis of single-label and multilabel learning [22, 23]. In a multimarket scenario, if a sample is related to multiple markers, the importance of these markers to the sample will generally be different, and the marker distribution is a marker form that describes the importance of different markers to the same sample. Label distribution learning is a machine learning method that takes label distribution as the learning target and has been applied in many fields. Author proposed a deep label distribution learning algorithm combining convolutional neural network and label distribution learning to estimate age by face, and Author uses wheel of emotions to automatically identify the user's emotional state from the text. Author proposed an algorithm based on multivariate label distribution to detect head pose [24, 25]. However, it has not yet been reported for the auxiliary diagnosis of brain diseases. This study aimed to identify particular qualities that aid in the automation of the diagnostics, as well as evaluating and contrasting various machine learning techniques [26]. The functional connectivity structure acquired from resting-state MRI that was being used to construct the auto-encoder that is semi-supervised for autism diagnosis in this research is proposed [27].

### 2.2. Marker Enhancements

Label distribution learning requires that the training data contain label distribution information. However, in real life, people often label samples in the form of single-label or multilabel, making it difficult to directly obtain label distribution information. Nonetheless, the labels of these data still contain relevant information about the distribution of the labels. Marker enhancement enhances the supervised information of samples through the implicit correlation between different sample markers, thereby achieving better results in marker distribution learning [28]. For example, the authors proposed tag augmentation as an auxiliary algorithm for tag distribution learning, which is used to mine the implied tag importance information in the training set, promote the original logical tag to tag distribution, and assist tag distribution learning. The authors proposed label-enhanced multilabel learning to reconstruct latent label importance information from logical labels to improve the performance of label distribution learning [29].

## 3. Cost-Sensitive Marker Distribution Learning for ASD-Aided Diagnosis

### 3.1. Symbolic Representation

The main symbols in this paper are expressed as follows: Use *x*_*i*_ ∈ *R*^*q*^ to represent the *i*^th^ sample, where *q* represents the dimension of the feature vector; *X* = [*x*_1_, *x*_2_, ⋯, *x*_N_] ∈ R^q×N^; *l*_*i*_=[*l*_*i*_^1^, *l*_*i*_^2^,……,*l*_*i*_^*K*^,]^*T*^ represents the logical token corresponding to *x*_*i*_, where *K* represents the number of possible tokens; and *l*_*i*_^*j*^ ∈ {0, 1}. Similarly, *d*_*i*_=[*d*_*i*_^1^, *d*,……,*d*_*i*_^*K*^,]^*T*^ ∈ *R*^*K*^ represents the label distribution of the *i*^th^ sample, where *d*_*i*_^*j*^ ∈ [0, 1] represents the *j*^th^ value of the label distribution of the *i*^th^ sample, satisfying ∑_*j*=1_^*K*^*d*_*i*_^*j*^=1, *D* = [*d*_1_,*d*_2_,⋯,*d*_N_] ∈ *R*^*K* × *N*^.

### 3.2. Proposed Methodology

**Figure 1:**
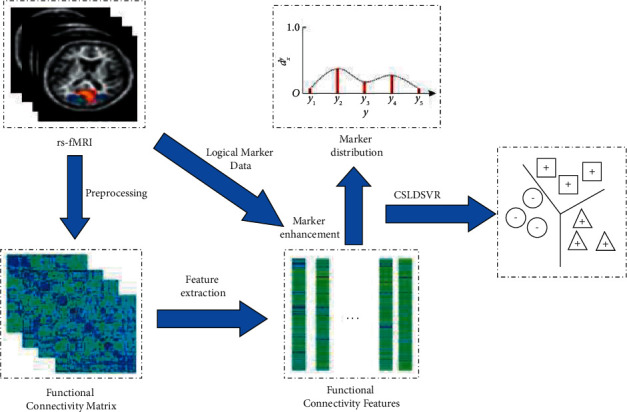
Proposed label distribution support vector regression for cost sensitivity.

The label distribution learning algorithm for multiclass autism auxiliary diagnosis proposed in this paper is shown in Figure 1. First, the rs-fMRI images are preprocessed, and the functional connectivity matrix is constructed on this basis, and the functional connectivity feature vector of each sample is obtained based on the functional connectivity matrix. At the same time, combining the logical marker data and functional connectivity features for marker enhancement, the marker distribution form of the sample is obtained. Finally, a cost-sensitive label distribution learning model is carried out to obtain a multi classification model for the auxiliary diagnosis of autism.

### 3.3. Marker Distribution Mechanism

Label distribution learning describes the degree of correlation between each label and sample by introducing a descriptive degree, so it can obtain richer semantic information from the data than multilabel and more accurately express the relative importance difference of multiple labels of the same sample. However, the basic requirement of labeled distribution learning is to have labeled distributed datasets, which is often difficult to meet in reality. The marker distribution data can be obtained by transforming a given multimarket form sample by a marker enhancement method. The label enhancement method based on FCM (fuzzy C-means) and fuzzy operation is adopted [30]. The basic idea is as follows:

(1) Use FCM to divide N samples into *p* fuzzy clusters, and find the center of each cluster, so that the sum of the weighted distances from all training samples to the cluster center is the smallest. Equation (1) lists the specific weighted distance formula:
  (1) $$\begin{array}{c} m_{x_{i}}^{k}=\frac{1}{{\sum_{j=1}^{P}\left(\text{Dist}\left(x_{i},\mu_{k}\right)/\text{Dist}\left(x_{i},\mu_{j}\right)\right)}^{1/\beta -1}}. \end{array}$$
Among them, *m*_*x*_*i*__^*k*^ represents the membership degree of the *i*^th^ sample to the *k*^th^ cluster center, *μ*_k_ represents the *k*^th^ cluster center, *β* is a fuzzy factor greater than 1, Dist (∗, ∗) represents the distance measure, and each sample the membership degree represents the strength of the association between the sample and the cluster. The clustering result of traditional FCM is greatly affected by the initial value and cannot ensure convergence to the global optimal solution, but in label enhancement, the clustering result of FCM is only used as a transitional bridge. Although the clustering result fluctuates, however, it has little effect on the results of label enhancement, and the gaps between the Chebyshev distance and the KL divergence (Kullback–Leibler divergence) of the results of multiple label enhancements are both below 10^−6^.
(2) Construct an association matrix A between markers and clusters. The elements in the matrix represent the degree of association between markers and clusters. The calculation method of the association matrix is as follows:
  (2) $$\begin{array}{c} A_{j}=A_{j}+m_{x_{i}}^{k}\;,\text{ if }l_{i}^{j}=1. \end{array}$$
In the formula, *A*_*j*_ is the *j*^th^ row of the matrix and *A*_*j*_ is the sum of the membership degree vectors of the samples of the *j*^th^ class. After the rows are normalized, the association matrix A can be regarded as a fuzzy relationship matrix of clustering and labeling.
(3) According to the fuzzy logic reasoning mechanism, the fuzzy synthesis operation is performed on the association matrix and the membership degree, and the membership degree of the sample to the label is obtained [31]. After normalization, it is the label distribution.

The marker enhancement based on FCM and fuzzy operation introduces cluster analysis as a bridge. Through the compound operation between the membership degree of the sample to the cluster and the membership degree of the cluster to the marker, the membership degree of the sample to the marker, that is, the marker, is obtained distributed. In this process, the topological relationship of the sample space is mined through fuzzy clustering, and this relationship is projected to the label space through the association matrix, so that the simple logical labeling generates richer semantic information and transforms it into a label distribution.

## 4. Evaluation of Proposed Methodology

### 4.1. Evaluation Metrics

This paper uses both the evaluation metric of the label distribution and the evaluation metric of the multiclassification task for algorithm evaluation. All evaluation indicators and calculation formulas are shown in Table 1. The first six are evaluation indicators for labeled distribution learning, and the last two are evaluation indicators for multiclassification tasks. “↑” after the index name means that the larger the value, the better the algorithm effect; with “↓,” the smaller the value, the better the algorithm effect.

In Table 1, *P*_*j*_ is the precision of the *j*^th^ class, xnor is the XOR calculation, Dis is the distance, Sim is the similarity, and mAP is the macro-averaging precision.

### 4.2. Dataset Used

All rs-fMRI datasets used in this paper were obtained from the ABIDE website (Autism Brain Imaging Data Exchange, http://fcon_1000.projects.nitrc.org/indi/abide/).  Table 2 shows the composition of each type of sample in each dataset. Taking the NYU (New York University) dataset as an example, the data collection institution of the NYU dataset is New York University. During the collection process, the subjects remained in a still state and did not perform any actions. The specific parameters are shown in Table 2.

In Table 2, UM stands for the University of Michigan, KKI for the Kennedy Krieger Institute, Leuven for the University of Leuven, and UCLA for the University of California, Los Angeles.

Although brain regions are spatially isolated from each other, the neural activity between them influences each other. This paper uses the brain functional connectivity matrix between brain regions as a classification feature [32]. The calculation step (preprocessing step) of the functional connectivity matrix is as follows:

1. According to the resting-state functional magnetic resonance imaging data, use the DPARSF (data processing assistant for resting-state fMRI) tool to extract the average time-series signals of each brain region, calculate the Pearson coefficient between the brain regions, and obtain the functional connectivity matrix
2. Take each row of the functional connectivity matrix as the feature description of each brain region, take the upper triangular matrix of the functional connectivity matrix, and connect the rows in series to obtain the corresponding eigenvectors

### 4.3. Proposed Algorithm

The proposed CSLDSVR method is compared with six existing LDL algorithms and two multiclassification algorithms. Two multiclassification algorithms are decision tree and K-nearest neighbor (KNN), both of which are classic multiclassification algorithms [33, 34]. The six existing LDL algorithms are PT-SVM, PT-BAYES, AA-KNN, AA-BP (back propagation), SA-IIS (improved iterative scaling), and LDSVR, where “PT” stands for problem transformation, “AA” for algorithm adaptation, and “SA” for specialized algorithm [35, 36]. The specific description of the comparison algorithm is shown in Table 3.

The CSLDSVR algorithm proposed in this paper has four parameters, namely, the weight coefficient C, the type of kernel function, the size of the insensitive region *ε*, and the kernel bandwidth of the Gaussian kernel. The specific range of parameters is shown in Table 4. The results were calculated using ten-fold cross-validation. The specific operation steps are as follows: Randomly divide the dataset into 10 equal parts in each fold cross validation, and take 1 part as the test set and the remaining 9 parts as the training set. Repeat the above process 10 times, and take the average of the 10 results as the evaluation index.

### 4.4. Comparison of Mark Distribution Algorithms

Table 5 summarizes the experimental results of six labeled distribution learning algorithms and CSLDSVR on five different datasets, and the experimental results are recorded in the form of mean ± standard deviation. Among them, the bold is the best value of each indicator in different methods on the current dataset. Clearly, in comparison with the label distribution learning algorithm, CSLDSVR has shown excellent results in most cases, and it is more obvious on the UM, UCLA, and KKI datasets. Among the indicators of the labeled distribution, KL divergence is an indicator describing the difference between the two distributions, and the LDL algorithm used as a comparison uses KL divergence as the objective function. The KL divergence of the prediction result of CSLDSVR can be minimized. It shows that the label distribution predicted by the new algorithm is the closest to the real data distribution on the whole, which is better than the comparison algorithm.

**Figure 2:**
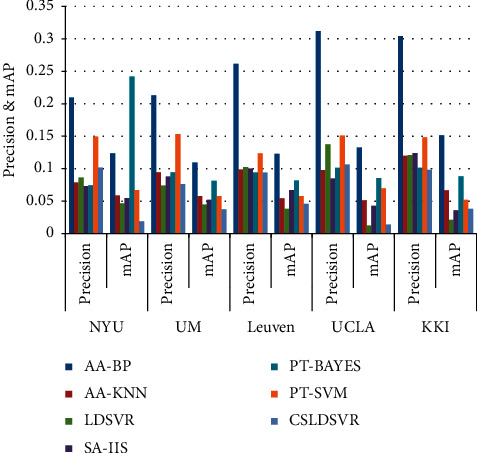
Comparative performance evaluation of CSLDSVR and label distribution algorithm.

Figure 2 summarizes the results of CSLDSVR and the marker distribution algorithm multiclass metrics precision and mAP; from the two most important multiclass metrics, CSLDSVR performs better. Some algorithms have a high accuracy rate but a low macro average because these algorithms do not consider the class imbalance problem, and the model classification is biased towards the majority class. CSLDSVR uses the kernel trick to solve the problem in a more discriminative feature space, and CSLDSVR considers the size of each class, which effectively solves the problem caused by class imbalance.

To verify the performance improvement of the cost-sensitive mechanism, the algorithm in this paper is compared with the LDSVR without the cost-sensitive mechanism. As shown in Table 5, in most cases, the learning effect of the algorithm CSLDSVR in this paper is better; in addition, the standard deviation of the results is basically maintained at a low level, that is, the stability of the algorithm is improved. However, LDSVR does not introduce a cost-sensitive mechanism, and the standard deviation of the results obtained by the algorithm is large and fluctuating. For example, the standard deviation of the Canberra indicators in UCLA and KKI exceeds 0.1.

### 4.5. Multiclass Comparison Experiment

Table 6 shows the comparison results of precision and mAP metrics of CSLDSVR and two classical multiclassification algorithms, decision tree and KNN, on five datasets. Among them, the bold is the best value of the corresponding indicator in different methods on the current dataset. Observing the experimental results of the KNN method, it can be found that the mAP of the KNN method appears 0.333 3 times, this is because KNN is too biased towards the majority class, and there is an extreme case of classifying all samples into the majority class. In the case of high-dimensional imbalance of autism neuroimaging data, traditional multiclassification algorithms are prone to fall into the dimensional trap or bias towards the majority class. The algorithm CSLDSVR in this paper solves the above problems by using kernel skills and cost-sensitive mechanisms and achieves better results. Good classification model. The cost-sensitive mechanism reduces the overall misclassification cost by increasing the misclassification cost of the minority class and reducing the misclassification cost of the majority class and makes the model avoid leaning towards the majority class. In other words, the cost-sensitive mechanism is based on the original standard cost loss function, adding some constraints and weight constraints, so that the final model is biased towards another minority class that is more concerned in practical applications. This paper achieves the purpose of different misjudgment costs for different categories by introducing 1Nj. In theory, this can avoid the tendency of the algorithm model to the majority class and improve the prediction accuracy for the minority class [37]. In the experiment, the experimental results in Table 6 also verify this theory, and in most cases, the stability of the algorithm has also been improved, and the standard deviation of the experimental results is small.

### 4.6. Effect of Parameters

**Figure 3:**
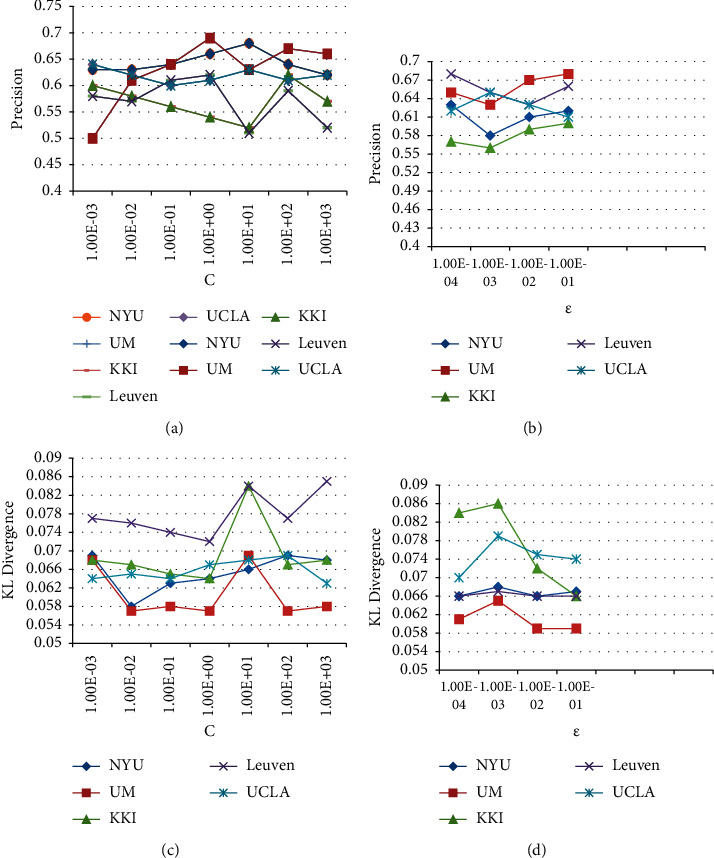
Changes of evaluation indicators. (a) Impact of C on precision, (b) impact of *ε* on precision, (c) impact of C on KL, and (d) impact of *ε* on KL.

In this section, we study the effect of parameter changes on the performance of the algorithm CSLDSVR.  Figure 3 shows the changes of the evaluation indicators precision and KL divergence when the parameters C and *ε* take different values on five different datasets. Comparing and studying two graphs of the same parameter and different indicators, such as Figures 3(a) and 3(c), it can be found that the curve trend of the same dataset is basically opposite, and the point where precision takes the maximum value is generally the same as the KL divergence is the minimum value, which also corresponds to the previous analysis of KL divergence, indicating that when the KL divergence is small, the label distributions of the two are more similar, and the classification results are more accurate.

It is found that for different datasets, the parameter values for obtaining the optimal solution are not the same, which also shows that in the diagnosis of autism, the data distribution of different data centers is different, and the parameters for building the model should also be different. Moreover, it is found that for a dataset with fewer samples, the result is more sensitive to the change of the parameters, such as for the KKI dataset with only 48 samples, the fluctuation is the largest when the parameter value changes.

It can be seen that the parameters of the CSLDSVR algorithm should be based on the characteristics of the dataset, and the corresponding parameter values should be set to build a model. If the parameter settings are reasonable, CSLDSVR can overcome the high dimensionality and category imbalance of the autism dataset. Thus, the whole section contains the strategy of how ASD detection is done by evaluating several strategies such as SVR and LDL by considering certain parameters. Therefore, the label distribution learning approach overcomes high-dimensional feature classification issues by mapping samples to the feature space and then diagnosing multiclass ASD. This technique outperforms previous methods in terms of classification performance and accuracy, as well as resolving the issue of unbalanced data in ASD diagnosis.

## 5. Conclusion

This research presents a cost-sensitive marker distribution to enable an ASD-aided diagnostic approach for vector regression based on functional connectivity characteristics collected from rs-fMRI. Since ASD patients' brain function differs from that of healthy persons,so rs-fMRI is a useful method for capturing brain activity. In this study, researchers have introduced the label distribution learning that solves the label noise problem in multiclassification ASD diagnosis. Furthermore, the new technique have been implemented, which provides class balancing and in addition balances the effect of the majority and minority classes on the objective function using the labeled distribution support vector regression method. The new method employed in this study effectively solves the imbalanced data problem in ASD diagnosis by overcoming the imbalance of the influence of the majority and minority classes on the results obtained in the paper. Besides, it presents a cost-sensitive approach to correct sample imbalance and uses a support vector regression (SVR)-based method to remove label noise. The label distribution learning approach overcomes high-dimensional feature classification issues by mapping samples to the feature space and then diagnosing multiclass ASD. The overall result obtained in this technique outperforms previous methods in terms of classification performance and accuracy, as well as resolves the issue of unbalanced data in ASD diagnosis. However, the improved model is still biased towards the majority class to some extent, and the imbalanced data problem should be improved further as a future study. Researchers can further try to improve the data sampling method or use the synthetic minority sample method, etc. as future prespective. However, relatively high-level distances must also be introduced, which necessitates more prior knowledge. Since prior knowledge is no longer used, the Euclidean distance is used instead. Other advanced distances have their set of benefits that will be refined in future research.

## Figures and Tables

**Table 1:** Evaluation measures.

	Index	Formula
Mark distribution metrics	Chebyshev↓	${\text{Dis}}_{1}=\underset{j\in \left[1,K\right]}{\max}\left|d_{i}^{j}-{\hat{d}}_{i}^{j}\right|$
	KL↓	${\text{Dis}}_{2}=\sum_{j=1}^{K}d_{i}^{j}\ln d_{i}^{j}/{\hat{d}}_{i}^{j}$
	Clark↓	${\text{Dis}}_{3}=\sqrt{\sum_{j=1}^{K}{\left(d_{i}^{j}-{\hat{d}}_{i}^{j}\right)}^{2}/{\left(d_{i}^{j}+{\hat{d}}_{i}^{j}\right)}^{2}}$
	Canberra↓	${\text{Dis}}_{4}=\sum_{j=1}^{K}\left|d_{i}^{j}-{\hat{d}}_{i}^{j}\right|/\left|d_{i}^{j}\right|+\left|{\hat{d}}_{i}^{j}\right|$
	Intersection↑	${\text{Sim}}_{1}=\sum_{j=1}^{K}\min\left(d_{i}^{j},{\hat{d}}_{i}^{j}\right)$
	Cosine↑	${\text{Sim}}_{2}=d_{i}\cdot {\hat{d}}_{i}/\left|d_{i}\right|\cdot \left|{\hat{d}}_{i}\right|$

Multiclass metrics	Precision	$P=1/N\sum_{i=1}^{N}\text{xnor}\left(l_{i},{\hat{l}}_{i}\right)$
	mAB	*mAB*=1/*N*∑_*i*=1_^*N*^*p*_*j*_

**Table 2:** Statistics of datasets.

Dataset	Number of samples	Normal	Autism	Asperger's syndrome
NYU	175	104	56	24
UM	140	73	54	16
KKI	52	37	7	8
Leuven	104	64	23	17
UCLA	83	51	18	14

**Table 3:** Comparison algorithms.

Comparison algorithm name	Description of the comparison algorithm
PT-SVM	Based on the problem-transformed SVM
PT-BAYES	BAYES-based gauss distribution
AA-*K*NN	The algorithm-based KNN
AA-BP	The algorithm-based BP neural network uses the softmax activation output as the predicted label distribution
SA-IIS	IIS based on dedicated algorithm uses an improved iterative scaling algorithm to optimize the objective function
LDSVR	LDSVR based on a dedicated algorithm
Decision tree	An instance-based inductive learning method
*K*NN	An instance-based classification method

**Table 4:** Range of parameters.

Parameter name	Parameter range
Weight factor	0.001, 0.01, 0.1, 1, 10, 100, 1000
Type of kernel function	Linear kernel, polynomial kernel, Gaussian kernel
Insensitive area size	0.0001, 0.001, 0.01, 0.1
The kernel bandwidth of the Gaussian kernel	0.01, 0.1, 1, 10, 100

**Table 5:** Comparative performance evaluation of CSLDSVR and LDL algorithms.

Evaluation metrics	Algorithm	NYU	UM	Leuven	UCLA	KKI
**Chebyshev↓**	AA-BP	0.223 7 ± 0.035 6	0.218 4 ± 0.045 8	0.248 0 ± 0.044 6	0.250 6 ± 0.053 5	0.254 7 ± 0.052 9
	AA-*K*NN	0.144 1 ± 0.011 6	0.154 0 ± 0.021 1	0.157 9 ± 0.026 5	0.142 6 ± 0.031 3	0.157 2 ± 0.029 5
	LDSVR	0.150 1 ± 0.024 3	0.140 0 ± 0.012 8	0.162 9 ± 0.034 4	0.169 4 ± 0.053 4	0.160 2 ± 0.057 0
	SA-IIS	0.147 8 ± 0.011 8	0.153 5 ± 0.023 7	0.174 8 ± 0.021 4	0.145 8 ± 0.032 9	0.162 7 ± 0.049 5
	PT-BAYES	0.381 8 ± 0.111 9	0.205 7 ± 0.009 5	0.206 9 ± 0.007 8	0.213 5 ± 0.009 9	0.215 4 ± 0.008 1
	PT-SVM	0.200 5 ± 0.041 2	0.188 5 ± 0.042 3	0.183 1 ± 0.040 1	0.195 8 ± 0.033 0	0.182 2 ± 0.058 9
	CSLDSVR	**0.141 3** ± **0.016 2**	**0.135 2** ± **0.023 6**	**0.140 2** ± **0.024 4**	**0.138 6** ± **0.038 4**	**0.126 7** ± **0.034 9**

**Cosine↑**	AA-BP	0.873 1 ± 0.034 4	0.881 8 ± 0.035 6	0.862 2 ± 0.049 8	0.839 9 ± 0.057 8	0.843 7 ± 0.058 6
	AA-*K*NN	0.935 4 ± 0.009 6	0.928 6 ± 0.017 3	0.927 4 ± 0.020 8	0.929 7 ± 0.022 4	0.913 0 ± 0.024 4
	LDSVR	0.937 7 ± 0.019 1	0.944 8 ± 0.013 3	**0.932 5** ± **0.029 2**	0.928 5 ± 0.052 0	0.932 6 ± 0.047 4
	SA-IIS	**0.940 7** ± **0.009 3**	0.934 4 ± 0.016 7	0.920 5 ± 0.016 0	0.939 5 ± 0.020 3	0.924 6 ± 0.042 5
	PT-BAYES	0.798 5 ± 0.071 3	0.915 6 ± 0.006 2	0.915 1 ± 0.005 3	0.910 4 ± 0.006 9	0.909 2 ± 0.005 7
	PT-SVM	0.898 7 ± 0.038 5	0.904 3 ± 0.042 8	0.914 5 ± 0.030 9	0.897 4 ± 0.036 5	0.906 8 ± 0.045 8
	CSLDSVR	0.940 5 ± 0.012 1	**0.947 3** ± **0.018 3**	0.923 4 ± 0.025 5	**0.942 8** ± **0.036 8**	**0.936 3** ± **0.029 4**

**Clark↓**	AA-BP	0.468 1 ± 0.064 8	0.461 3 ± 0.099 0	0.517 0 ± 0.083 8	0.537 1 ± 0.110 1	0.542 7 ± 0.104 6
	AA-*K*NN	0.263 1 ± 0.020 3	0.282 2 ± 0.036 7	0.287 3 ± 0.047 3	0.261 3 ± 0.053 5	0.283 2 ± 0.053 9
	LDSVR	0.272 9 ± 0.036 4	0.255 7 ± 0.021 8	0.287 2 ± 0.062 6	0.295 6 ± 0.092 0	0.281 9 ± 0.100 8
	SA-IIS	0.266 3 ± 0.019 1	0.278 8 ± 0.039 7	0.311 3 ± 0.033 6	0.262 3 ± 0.055 5	0.293 9 ± 0.088 0
	PT-BAYES	0.893 6 ± 0.359 8	0.352 0 ± 0.014 5	0.352 3 ± 0.012 7	0.363 6 ± 0.016 2	0.366 3 ± 0.013 3
	PT-SVM	0.358 0 ± 0.070 2	0.348 1 ± 0.075 8	0.325 3 ± 0.065 5	0.350 5 ± 0.056 1	0.328 7 ± 0.098 1
	CSLDSVR	0.261 6 ± 0.032 1	0.246 3 ± 0.037 6	**0.253 9** ± **0.041 8**	**0.248 4** ± **0.062 6**	**0.233 4** ± **0.061 8**

**Intersection↑**	AA-BP	0.776 3 ± 0.035 6	0.781 6 ± 0.045 8	0.752 0 ± 0.044 6	0.749 4 ± 0.053 5	0.745 3 ± 0.052 9
	AA-*K*NN	0.855 9 ± 0.011 6	0.846 0 ± 0.021 1	0.842 1 ± 0.026 5	0.857 4 ± 0.031 3	0.842 8 ± 0.029 5
	LDSVR	0.849 9 ± 0.024 3	0.860 0 ± 0.012 8	0.837 1 ± 0.034 4	0.830 6 ± 0.053 4	0.839 8 ± 0.057 0
	SA-IIS	0.852 2 ± 0.011 8	0.846 5 ± 0.023 7	0.825 2 ± 0.021 4	0.854 2 ± 0.032 9	0.837 3 ± 0.049 5
	PT-BAYES	0.618 2 ± 0.111 9	0.794 3 ± 0.009 5	0.793 1 ± 0.007 8	0.786 5 ± 0.009 9	0.784 6 ± 0.008 1
	PT-SVM	0.799 5 ± 0.041 2	0.811 5 ± 0.042 3	0.816 9 ± 0.040 1	0.804 2 ± 0.033 0	0.817 8 ± 0.058 9
	CSLDSVR	0.858 7 ± 0.041 5	0.864 8 ± 0.023 6	0.859 8 ± 0.024 4	0.861 4 ± 0.038 4	0.873 3 ± 0.034 9

**KL↑**	AA-BP	0.166 7 ± 0.042 9	0.161 2 ± 0.051 7	0.192 0 ± 0.069 3	0.222 2 ± 0.089 8	0.227 9 ± 0.076 4
	AA-*K*NN	0.068 5 ± 0.010 1	0.076 0 ± 0.018 4	0.076 6 ± 0.022 1	0.074 6 ± 0.023 2	0.093 2 ± 0.026 6
	LDSVR	0.066 5 ± 0.019 9	0.059 3 ± 0.014 6	0.070 3 ± 0.032 3	0.074 9 ± 0.062 5	0.071 1 ± 0.049 8
	SA-IIS	0.063 9 ± 0.009 3	0.069 8 ± 0.017 8	0.083 7 ± 0.016 6	0.063 9 ± 0.021 0	0.080 0 ± 0.044 1
	PT-BAYES	0.492 9 ± 0.251 0	0.087 9 ± 0.006 7	0.088 0 ± 0.006 1	0.093 5 ± 0.008 0	0.094 8 ± 0.006 6
	PT-SVM	0.108 1 ± 0.041 2	0.105 5 ± 0.047 6	0.090 6 ± 0.032 9	0.110 4 ± 0.040 5	0.100 3 ± 0.048 1
	CSLDSVR	0.060 3 ± 0.041 5	0.056 7 ± 0.019 5	0.069 9 ± 0.024 0	0.060 1 ± 0.046 1	0.068 2 ± 0.030 1

**Table 6:** Performance evaluation for multiclassification algorithms.

Dataset	Decision tree		KNN		CSLDSVR	
	Precision	mAP	Precision	mAP	Precision	mAP
NYU	0.548 8 ± 0.1423	0.409 3 ± 0.0703	0.614 4 ± 0.1525	0.364 7 ± 0.0527	**0.655 4** ± **0.0571**	**0.451 7** ± **0.0398**
UM	0.576 7 ± 0.1325	0.385 9 ± 0.0872	0.528 5 ± 0.1214	0.374 0 ± 0.0861	**0.701 4** ± **0.0708**	**0.497 1** ± **0.1250**
Leuven	0.617 1 ± 0.2261	0.424 2 ± 0.2086	0.608 5 ± 0	0.333 3 ± 0	**0.617 6** ± **0.0725**	**0.448 2** ± **0.0861**
UCLA	0.605 2 ± 0.1833	0.442 0 ± 0.2086	0.654 3 ± 0	0.333 3 ± 0	**0.665 2** ± **0.1504**	**0.443 4** ± **0.1659**
KKI	0.559 8 ± 0.2567	0.395 4 ± 0.2941	0.646 5 ± 0	0.333 3 ± 0	**0.687 5** ± **0.1237**	**0.447 6** ± **0.1016**

## Data Availability

The data shall be made available on request.
